# Viroporin Activity of the Foot-and-Mouth Disease Virus Non-Structural 2B Protein

**DOI:** 10.1371/journal.pone.0125828

**Published:** 2015-05-06

**Authors:** Da Ao, Hui-Chen Guo, Shi-Qi Sun, De-Hui Sun, To Sing Fung, Yan-Quan Wei, Shi-Chong Han, Xue-Ping Yao, Sui-Zhong Cao, Ding Xiang Liu, Xiang-Tao Liu

**Affiliations:** 1 State Key Laboratory of Veterinary Etiological Biology and OIE/National Foot and Mouth Disease Reference Laboratory, Lanzhou Veterinary Research Institute, Chinese Academy of Agricultural Sciences, Lanzhou, Gansu, China; 2 College of Veterinary Medicine, Sichuan Agricultural University, Ya’an, Sichuan, China; 3 School of Biological Sciences, Nanyang Technological University, Singapore, Singapore; Lady Davis Institute for Medical Research, CANADA

## Abstract

Viroporins are a family of low-molecular-weight hydrophobic transmembrane proteins that are encoded by various animal viruses. Viroporins form transmembrane pores in host cells via oligomerization, thereby destroying cellular homeostasis and inducing cytopathy for virus replication and virion release. Among the *Picornaviridae* family of viruses, the 2B protein encoded by enteroviruses is well understood, whereas the viroporin activity of the 2B protein encoded by the foot-and-mouth disease virus (FMDV) has not yet been described. An analysis of the FMDV 2B protein domains by computer-aided programs conducted in this study revealed that this protein may contain two transmembrane regions. Further biochemical, biophysical and functional studies revealed that the protein possesses a number of features typical of a viroporin when it is overexpressed in bacterial and mammalian cells as well as in FMDV-infected cells. The protein was found to be mainly localized in the endoplasmic reticulum (ER), with both the N- and C-terminal domains stretched into the cytosol. It exhibited cytotoxicity in *Escherichia coli*, which attenuated 2B protein expression. The release of virions from cells infected with FMDV was inhibited by amantadine, a viroporin inhibitor. The 2B protein monomers interacted with each other to form both intracellular and extracellular oligomers. The Ca^2+^ concentration in the cells increased, and the integrity of the cytoplasmic membrane was disrupted in cells that expressed the 2B protein. Moreover, the 2B protein induced intense autophagy in host cells. All of the results of this study demonstrate that the FMDV 2B protein has properties that are also found in other viroporins and may be involved in the infection mechanism of FMDV.

## Introduction

Foot-and-mouth disease (FMD) is a highly contagious disease in animals and is on the Office International Des Epizooties (OIE) list of notifiable animal diseases[1]. The causative agent of FMD is the foot-and-mouth disease virus (FMDV), which is a non-enveloped virus with icosahedral symmetry. The FMDV belongs to the *Aphthovirus* genus of the *Picornaviridae* family. It has a single-stranded, plus-sense RNA genome that consists of approximately 8,500 bases. The genome is divided into 3 regions, a 5’ non-coding region, a protein-coding region, and a 3’ non-coding region [2]. The protein-coding region can be further divided into the P1, P2, and P3 regions. The P1 region encodes four capsid proteins, and the P2 and P3 regions encode non-structural proteins, including the 2B protein. The research on FMDV non-structural proteins has increased over the last few years, but additional efforts are needed to obtain more information, especially on the 2B protein, which may act as a viroporin.

Viroporins are small, hydrophobic proteins encoded by a wide range of viruses [3]. In recent years, a growing number of viral proteins have been added to the viroporin family and have garnered significant interest because of their central role in the viral life cycle. Viroporins can oligomerize in host cell membranes to form hydrophilic pores that disrupt the physiological properties of host cells. Viroporins are typically composed of 60–120 amino acids and contain one or two highly hydrophobic domains that can form an amphipathic α-helix after insertion into the phospholipid bilayer. In addition to their common architecture, viroporins share common functions, such as modifying membrane permeability, disturbing the Ca^2+^ balance in host cells, and inducing autophagy and apoptosis after expression in cells [3–7].

The influenza A virus (IAV) M2 protein was the first protein to be studied as an ion channel [8, 9]. Subsequently, many viral proteins, including HIV-1 viral protein U (Vpu), the hepatitis C virus (HCV) p7protein, the classical swine fever virus (CSFV) p7protein, and the togavirus 6K protein, have been identified as members of the viroporin family [5]. Regarding the *Picornavirus* family of viruses, only the 2B proteins of poliovirus and coxsackie virus have been extensively studied. The 2B proteins of poliovirus and coxsackie virus contain two hydrophobic regions, and they can insert themselves into the membrane of the endoplasmic reticulum (ER) or the Golgi apparatus to modify cellular membrane permeability once they are expressed in host cells [10–12]. Additionally, these 2B proteins can disrupt the Ca^2+^ balance in host cells, inducing apoptosis [13, 14]. However, few reports are available on the 2B protein of FMDV, which belongs to the *Picornavirus* family.

To obtain more information on the FMDV 2B protein, we analyzed the sequence of this protein and predicted its structure. The results of this analysis indicated that the 2B protein of FMDV contains two hydrophobic regions and inserts itself into the membrane of the ER with its N- and C-termini oriented towards the cytosol. During expression in host cells, the 2B protein increases the membrane permeability of bacterial and mammalian cells and can increase the Ca^2+^ content in host cells, thereby inducing autophagy. Altogether, these results demonstrate that the FMDV 2B protein has the same properties as other viroporins, suggesting that the 2B proteins of picornaviruses may play the same role in virus infection.

## Materials and Methods

### Mammalian cells and *Escherichia coli*

Baby hamster kidney 21 (BHK-21) cells and H1299 cells were purchased from China Center for Type Culture Collection (CCTCC, Wuhan, China). H1299-LC3 cells demonstrating stable expression of LC3 were established according to previously report [15]. Briefly, the H1299 cells were transfected with the pEGFP-LC3 plasmid, and the stable transfectants were selected by G418. The GFP-LC3 protein expression was analyzed by Western blot. The fluorescent signals were detected by inverted fluorescence microscope. All of the cell lines were maintained in Dulbecco’s modified Eagle’s medium (DMEM, Gibco, California, USA) supplemented with 10% fetal calf serum (FBS, Gibco, California, USA) and1% penicillin-streptomycin (Invitrogen, California, USA). The *Escherichia coli* C43(DE3)pLysS and BL21(DE3)pLysS strains were stored at -80°C.

### Construction of plasmids

The FMDV 2B gene was amplified by polymerase chain reaction (PCR) from a vector that contained the full-length genome of the FMDV serotype Asia1 using specific primers. For topology analysis, different recombinant plasmids with various tags fused to the 2B gene at the N- or C-terminus were constructed using previously described methods[16]. The plasmids included pSUMO-2B, pEGFPN1-2B, pCS2-2B, pXJ-FLAG-2B with a FLAG epitope tag (DYKDDDDKS) connected to the N-terminal domain, pXJ-2B-HA with an HA epitope tag (YPYDVPDYA) connected to the C-terminal domain, and pXJ-2BΔ(amino acids 110–111) with a FLAG epitope tag inserted between the 110th and 111th amino acid of the 2B protein. All of the plasmids were verified using standard sequencing techniques.

The cDNA of human microtubule-associated proteins 1A/1B light chain 3B (LC3, NCBI RefSeq NM_022818.4) was amplified from cDNA of H1299 cells using the primers 5’-CCGGAATTCCATGCCGTCGGAGAAGAC-3’ and 5’-CGGGGTACCAACAATTCTAGAAGAGCTGCA-3’. The PCR product was digested with *EcoRI* and *KpnI* and inserted into the vector pEGFP-C1 (Clontech). The resulting plasmid was named pEGFP-LC3.

### Transmembrane domain prediction for the 2B protein

The 2B protein of the FMDV was analyzed using the TMpred (http://www.ch.embnet.org/software/TMPRED_form.html), DAS-TMfilter (http://mendel.imp.univie.ac.at/sat/DAS/), and PredictProtein (https://www.predictprotein.org/) programs.

### Subcellular localization of the 2B protein in BHK-21 cells

The plasmid pEGFPN1 and the recombinant plasmid pEGFPN1-2B were transfected into BHK-21 cells using Lipofectamine 2000 (Invitrogen, California, USA). At 12 hours post-transfection, both dishes of cells were washed with phosphate-buffered saline (PBS) 3 times. The ER organelle was stained with ER-Tracker Red (Beyotime, Jiangsu, China) according to the manufacturer’s instructions. The nucleus was stained with 4',6-diamidino-2-phenylindole (DAPI, Beyotime, Jiangsu, China,0.5 μg/mL). The cells were observed under a laser-scanning confocal microscope (LSCM, Leica SP8, Solms, Germany) at the wavelengths 340nm, 488nm, and 561nm.

### Membrane topology of the 2B protein in BHK-21 cells

The plasmids pXJ-FLAG-2B, pXJ-2B-HA, and pXJ-2BΔ(amino acids 110–111) were transfected into BHK-21 cells usingLipofectamine2000 (Invitrogen, California, USA). At 12 hours post-transfection, all of the cells were fixed with 4% paraformaldehyde at room temperature for 15 minutes and washed with PBS 3 times. Subsequently, the two groups of transfected BHK-21 cells were treated with Triton X-100 (0.01% [vol/vol]) or digitonin (0.01 mg/mL) at room temperature for 10 minutes. All of the cells were incubated with corresponding primary and secondary antibodies for 1 hour at 37°C. After washing, all of the cells were dyed with DAPI (0.5 μg/mL) and washed again with PBS 3 times. All of the samples were observed under a LSCM at the wavelengths 340nm and 488nm.

### Cytotoxicity of the 2B protein in *Escherichia coli*

The recombinant plasmids pSUMO and/or pSUMO-2B were transformed into the *Escherichia coli* BL21(DE3)pLysS and C43(DE3)pLysS strains, respectively. Positive bacterial clones were cultured overnight in TB medium with 10 μg/mL kanamycin at 37°C. Subsequently, the bacteria were diluted 100-fold in TB medium that contained similar antibiotics. When the OD600 of the cultures reached 0.6–0.7, 1 mM isopropyl-β-d-thiogalactoside (IPTG) was added. The bacterial solution was collected at different time. The OD600 of the samples was measured, and a Western blot analysis was performed to assess the expression level of the 2B protein.

### Effect of amantadine on the release of FMDV

BHK-21 cells were grown to a density of 80% confluence, infected with FMDV (multiplicity of infection [MOI] = 0.1) for 1 hour, and then washed twice with PBS. Next, the cells were incubated in growth medium with the indicated concentrations of amantadine for 4 hours. The supernatant was collected, and the amount of viral progeny released into the supernatant was determined based on the TCID50.

### Oligomerization of the 2B protein

The 2B protein was expressed and purified after transformation of the pSUMO-2B plasmid into the *Escherichia coli* C43(DE3)pLysS strain. A 20 μL aliquot of the solution that contained the purified 2B protein was added to a glutaraldehyde solution at different concentrations to obtain the final concentrations 0 mM, 0.1 mM, 0.5 mM, 1 mM, 1.5 mM, 2 mM, and 3 mM. The solution was rotated on a rotator device at 4°C for 2 hours and evaluated by Western blot analysis with a specific antibody.

The recombinant plasmids pXJ-FLAG-2B and pXJ-2B-HA were transfected into BHK-21 cells simultaneously or alone. At 12 hours post-transfection, the cells were collected for co-immunoprecipitation according to the instructions of the Pierce HA-Tag IP/Co-IP Kit (Thermo, Massachusetts, USA). The co-IPed proteins were detected via western blot with the corresponding antibodies

### Changes in cellular Ca^2+^ concentrations

The pXJ-2B-HA plasmid was transfected into BHK-21 cells, and untreated cells were used as a negative control. At 12 hours post-transfection, the cells were stained with propidium iodide (PI) and Fluo-3 AM following the protocol provided by the manufacturer. The samples were analyzed using flow cytometry at the wavelengths 561 nm and 535 nm.

### Autophagy induced in host cells expressing the 2B protein

BHK-21 cells were cultured and subjected to different treatments. Cells that were not treated or were transfected with the pXJ vector were used as a negative control, and cells treated with rapamycin (200 nM) were used as a positive control. The cells were collected in cell lysis buffer that contained a proteinase inhibitor at 12 hours post-transfection. Western blot analysis was performed with specific antibodies to detect the LC3-Iand LC3-II expression levels.

For the indirect immunofluorescence assay (IFA), cells that were not treated or were transfected with pCS2 were used as a negative control, and cells treated with rapamycin (200 nM) were used as a positive control. The cells in the experimental group were transfected with the pCS2-2B plasmid. At 12 hours post-transfection, the cells in each group were fixed with 4% paraformaldehyde and incubated with specific antibodies. Fluorescence was detected using a LSCM at the wavelengths 340 nm, 488 nm, and 561 nm.

The H1299 cell line, which stably expresses GFP-LC3, was transfected with the pCS2-2B plasmid. Cells that were not treated or were transfected with pCS2 were used as a negative control, and cells treated with rapamycin (200 nM) were used as a positive control. At 12 hours post-transfection, the cells in each group were fixed with 4% paraformaldehyde, and the fluorescence was detected using a LSCM at the wavelengths 340 nm, 488 nm, and 561 nm.

### Statistical analysis

Differences between the blank control group and the target protein expression group were analyzed using the SPSS Statistics 19.0 software. **A** one-way ANOVA followed by a Least Significance Difference (LSD) test was applied to compare the expression of the 2B protein in *Escherichia coli* BL21(DE3)pLysS and C43(DE3)pLysS and to compare the numbers of GFP-LC3 puncta in cells expressing 2B protein and controls. At test was used to analyze the significance of the effect of amantadine on cells infected with FMDV and the effect of 2B protein expression on the cellular Ca^2+^ concentration. The level of significance for all statistical tests was set at 0.05 (p < 0.05).

## Results

### Transmembrane domain of the FMDV 2B protein

**Fig 1:**
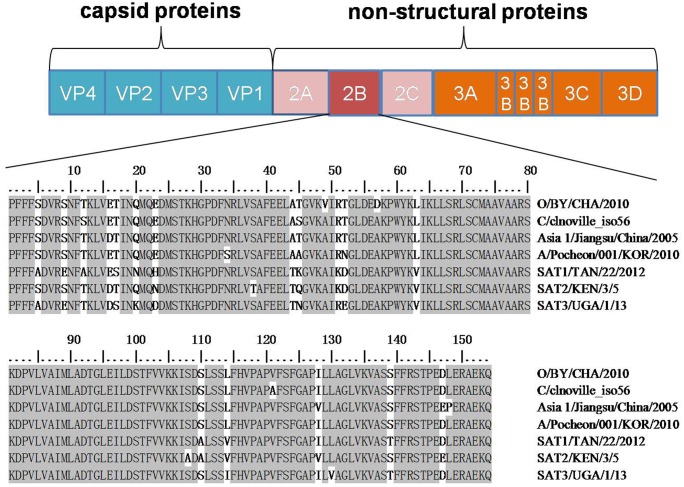
A schematic representation of the FMDV proteins and a multiple alignment of the 2B protein among FMDV serotypes. One isolate of each serotype of the 2B protein sequences was listed in the alignment. The sequences labeled in red represent the two transmembrane domains predicted by the online software.

Computer-assisted topology predictions are useful for experimental studies on transmembrane proteins. A bioinformatic prediction of the FMDV 2B protein was conducted using three popular prediction methods, which provide online predictions of the characteristics of the protein of interest based on the submitted amino acid sequence. This analysis revealed that the FMDV 2B gene contains 462 bases that encode 154 amino acids. Sequence analyses of the 2Bprotein predicted that there are two putative transmembrane domains in the peptide, from aa83-104 and from aa119-137, which are connected by a short basic segment (Fig 1).

### Subcellular localization of the FMDV 2B protein in BHK-21 cells

**Fig 2:**
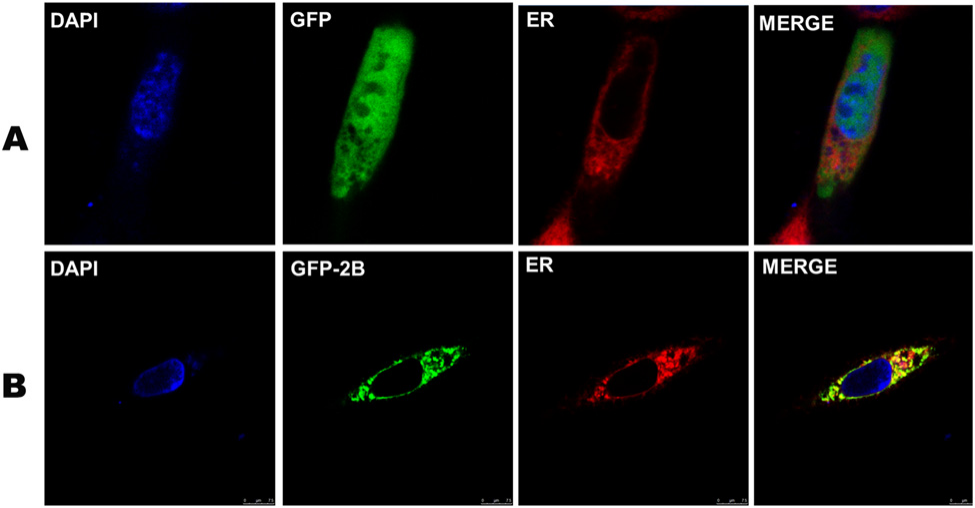
The subcellular localization of the FMDV 2B protein in BHK-21 cells. BHK-21 cells were transfected with pEGFPN1 (A) and pEGFPN1-2B (B) and evaluated using a LSCM. The blue fluorescence represents the nucleus, the green fluorescence represents the GFP protein or GFP-2B protein, and the red fluorescence represents the ER.

Several studies have indicated that most viroporins are located intracellularly[3]. Further studies have revealed that the 2B proteins of poliovirus and coxsackie virus are located in the ER or the Golgi apparatus. Additionally, Moffat *et al*. studied the effects of FMDV 2BC and 3A on the early secretion pathway in infected cells and found that they block the delivery of proteins to the cell surface by interacting with the ER [17]. They also proposed that 2B is associated with the ER in Vero cells. To determine the subcellular localization of the FMDV 2B protein in BHK-21 cells, the recombinant plasmid pEGFPN1-2B, which expresses the GFP-2B fusion protein, was constructed, and the eukaryotic expression vector pEGFPN1 was used as a negative control. ER-Tracker Red, a red fluorescent probe, was used to stain the ER with a specific fluorescence in viable cells. As shown in Fig 2A, the green and red fluorescence did not co-localize in the cells that expressed GFP. However, in the cells that expressed GFP-2B, the green fluorescent GFP protein co-localized with the red fluorescence of the ER, as shown in Fig 2B. This finding suggests that the FMDV 2B protein was mainly located in the ER of cells that overexpressed the protein. Our results are consistent with the findings reported by Moffat *et al*.

### Membrane topology of the FMDV 2B protein in BHK-21cells

**Fig 3:**
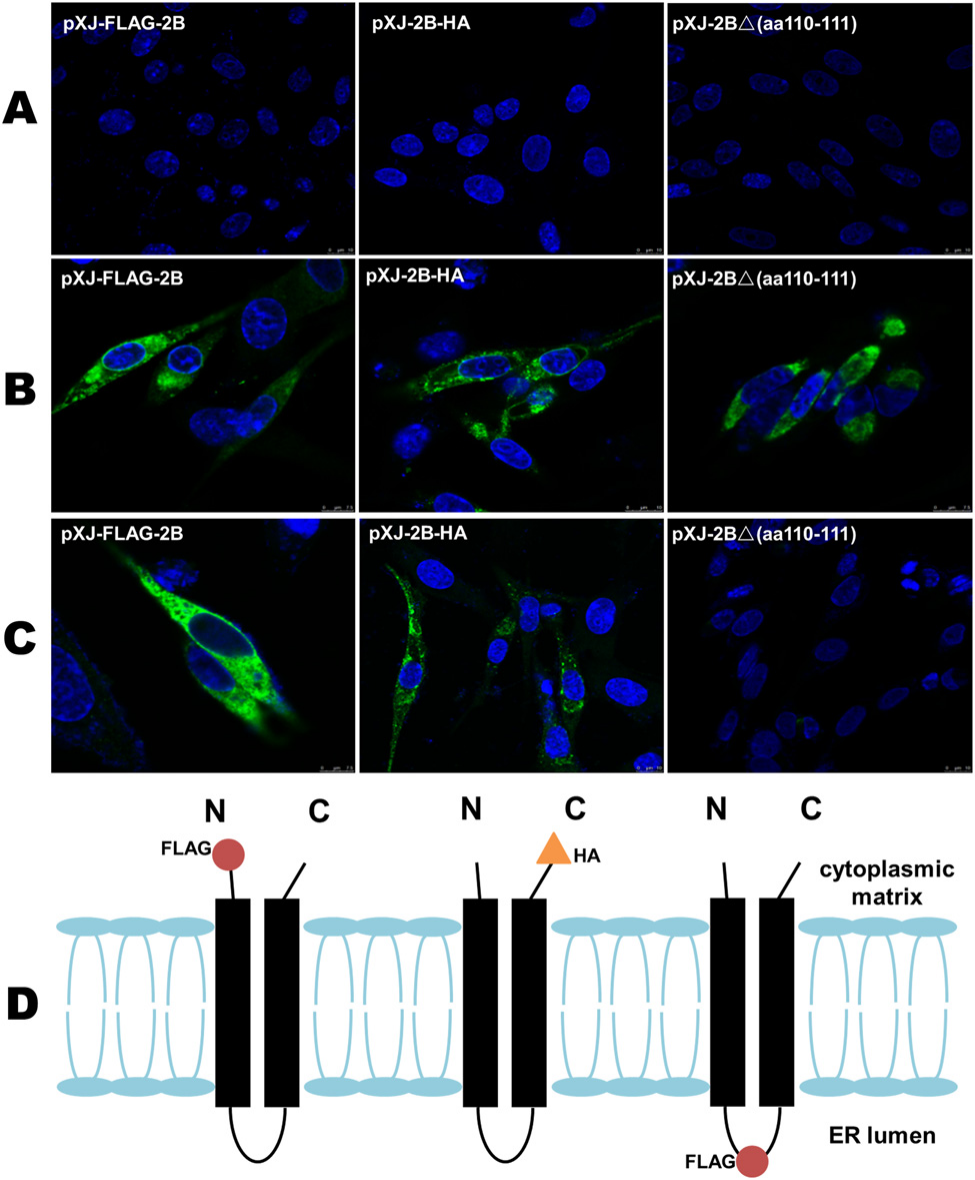
The topological structure of the FMDV 2B protein. Three groups of cells were transfected with pXJ-FLAG-2B, pXJ-2B-HA, or pXJ-2BΔ(110–111). (A) Cells cultured without any treatment. (B) Cells treated with Triton X-100. (C) Cells treated with digitonin. (D) A schematic representation of the predicted topology of the FMDV 2B protein in the ER membrane. The blue fluorescence represents the nucleus, and the green fluorescence represents the FLAG-2B protein or the 2B-HA protein.

Based on previous studies [16, 18] and the prediction of the transmembrane domain in this study, plasmids were constructed to confirm the topology of the FMDV 2B protein. These plasmids included pXJ-FLAG-2B, which expresses the 2B protein with a FLAG tag fused to the N-terminus; pXJ-2B-HA, which expresses the 2B protein with an HA tag fused to the C-terminus; and pXJ-2BΔ(aa 110–111), in which a FLAG tag was inserted between aa 110–111. After cells were transfected with these plasmids and subjected to different permeabilization treatments, the tags were detected via IFA using specific antibodies. As shown in Fig 3, no green fluorescence was observed in the untreated cells transfected with the different plasmids, suggesting that these specific antibodies could not pass through the intact plasma membrane into the cells (Fig 3A). After Triton X-100 treatment [19–21], green fluorescence was observed in the cells transfected with pXJ-FLAG-2B, pXJ-2B-HA, or pXJ-2BΔ(aa 110–111), suggesting that Triton X-100 completely permeated the plasma membrane and that the antibodies specifically detected the overexpressed 2B proteins with the corresponding tags (Fig 3B). However, after digitonin treatment [16, 22], green fluorescence was still observed in the cells transfected with pXJ-FLAG-2B or pXJ-2B-HA, but not in the cells transfected with pXJ-2BΔ(aa110-111) (Fig 3C). This finding suggests that the antibodies could not penetrate the ER membrane after treatment with 0.01 mg/mL digitonin to detect the FLAG tag between the two transmembrane domains. Furthermore, this finding also revealed that the FLAG tag inserted between the two transmembrane domains was located inside the ER membrane with a lumen orientation. These results confirmed that the FMDV 2B protein contains two transmembrane domains, with a cytosolic orientation of the N- and C-termini, as shown in Fig 3D. Therefore, the FMDV 2B protein may be a member of the class IIB viroporins according to its transmembrane topology.

### Cytotoxicity of the 2B protein in *Escherichia coli*

**Fig 4:**
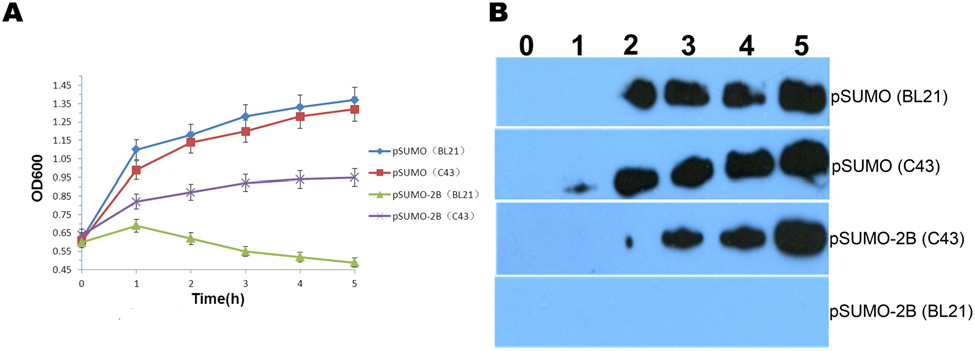
The cytotoxicity of the 2B protein in *Escherichia coli*. (A) The proliferation curves of the recombinant bacteria carrying different plasmids are presented. The pSUMO and pSUMO-2B plasmids were transfected into the *Escherichia coli* BL21(DE3)pLysS or C43(DE3)pLysS strain. The OD600 was determined at 0h, 1h, 2h, 3h, 4h, and 5h post-induction. (B) Expression of the 2B protein in the bacteria. The pSUMO and pSUMO-2B plasmids were transfected into the *Escherichia coli* BL21(DE3)pLysS or C43(DE3)pLysS strain. The products were analyzed by Western blot analysis with an anti-His antibody.

Several studies have investigated the cytotoxicity of viroporins in *Escherichia coli*, such as the IAV M2 protein [8], the poliovirus 2B protein [23] and the severe acute respiratory syndrome-associated coronavirus envelope protein [24]. To confirm the cytotoxicity of the FMDV 2B protein, two bacterial strains, BL21(DE3)pLysS and C43(DE3)pLysS, were used. *Escherichia coli* BL21(DE3)pLysS is a widely used impressionable strain; therefore, this strain was used to express the 2B protein as a control. The *Escherichia coli* C43(DE3)pLysS strain can improve the stability of plasmids during the expression of toxic recombinant proteins [25, 26]. After transformation of the two *Escherichia coli* strains with the pSUMO and pSUMO-2B plasmids, the expression of the 2B protein was induced using IPTG at different time points. As shown in Fig 4A, both the BL21(DE3)pLysS cells and the C43(DE3)pLysS cells that were transformed with the pSUMO plasmid grew well and demonstrated similar growth tendencies according to the obtained OD600 values. However, a dramatic decrease in cell density occurred in the BL21(DE3)pLysS cells that expressed the 2B protein. In contrast, the density of the C43(DE3)pLysS cells continued to increase. A comparison of the data between pSUMO(BL21) and pSUMO-2B(BL21) and between pSUMO(C43) and pSUMO-2B(C43), showed that there was a significant difference between pSUMO(BL21) and pSUMO-2B(BL21) (p = 0.000, p<0.01) and between pSUMO(C43) and pSUMO-2B(C43) (p = 0.000, p<0.01). Additionally, it also showed that there was a significant difference between pSUMO-2B(BL21) and pSUMO-2B(C43) (p = 0.000, p<0.01). However, there was no difference between pSUMO(BL21) and pSUMO(C43) (p = 0.502, p>0.05). These results suggest that the FMDV 2B protein, but not SUMO, is lytic in BL21(DE3)pLysS cells.

To further confirm whether the decreased growth of bacterial cells negatively affected the expression of the 2B protein, Western blot analysis with a specific antibody was used to detect the 2B protein level. Fig 4B shows that the SUMO protein was expressed in the bacterial cells at 2 hours post-induction. Additionally, the C43(DE3)pLysS cells transformed with the pSUMO-2B plasmid expressed the 2B protein at 2 hours post-induction. However, no specific band was detected in the lysed BL21(DE3)pLysS cells. These results are consistent with those presented in Fig 4A and further suggest that the 2B protein is toxic to BL21(DE3)pLysS cells.

### Effect of amantadine on the release of FMDV

**Fig 5:**
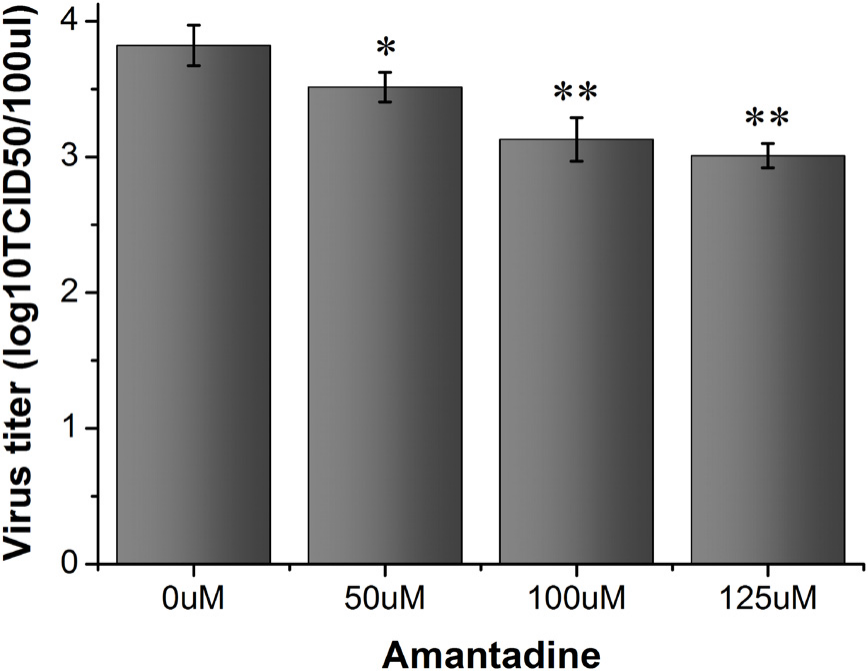
The effect of amantadine on the release of FMDV virions. BHK-21 cell cultures were infected with FMDV (MOI/0.1) and then treated with amantadine. The virus in the supernatant was collected at 4 hours post-infection. The virus titer was determined by TCID50. Asterisks indicate significant differences between the indicated samples (*P<0.05,** P<0.01).

Amantadine is an effective inhibitor of viroporins, including the IAV M2 protein [27, 28], the HCV p7 protein [29], and the C-terminal subunit of the p13 protein (p13-C) of GB virus B (GBV-B) [30]. As previously demonstrated, viroporins can promote the release of virions. To investigate the possible mechanism of virion release, the effects of amantadine on FMDV replication were evaluated [31]. BHK-21 cells were treated with or without amantadine for 30 minutes after infection with FMDV. The supernatant was collected at 4 hours post-infection, diluted from 10^-1^ to 10^-9^, and then added to fresh BHK-21 cells. The virus titer (TCID50) was determined after culturing the cells for 72 hours. Fig 5 shows that the virus titer decreased as the amantadine concentration increased, which suggests that amantadine inhibited the release of the virus from the cells and that viroporins may be involved in this process.

### Oligomerization of FMDV 2B protein monomers

Pore-forming activity is an important property of viroporins. The monomers of viroporins, such as the IAV M2 protein [32], the HCV p7 protein [33] and the CSFV p7 protein [16], can oligomerize to form pores. Furthermore, the 2B proteins of poliovirus and coxsackie virus also demonstrate pore-forming activity [34–36].

**Fig 6:**
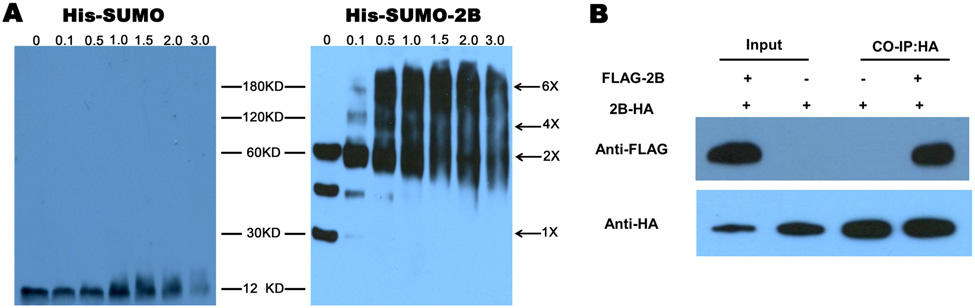
The pore-forming activity of the 2B protein. (A) Purified Sumo-2B protein was incubated with a glutaraldehyde cross-linker at the indicated concentrations (0, 0.1, 0.5, 1.0, 1.5, 2.0, and 3.0 mM). The monomers and oligomers of the 2B protein were detected by immunoblot analysis with an anti-His monoclonal antibody. (B) BHK-21 cells were transfected with pXJ-FLAG-2B or pXJ-2B-HA. The cell lysates were subjected to immunoprecipitation using anti-HA antibodies.

To determine whether the FMDV 2B monomers interact with each other *in vitro* and *in vivo*, oligomerization of the 2B protein was detected by Western blot analysis using a cross-linking agent. The 2B protein was expressed in the *Escherichia coli* C43(DE3)pLysS strain and purified from the supernatant of bacterial lysates. Then, glutaraldehyde, a widely used cross-linking agent [37], was used to cross-link the 2B oligomers. Fig 6A shows that the His-SUMO protein, which was used as a control, did not oligomerize in the presence of different concentrations of glutaraldehyde (left side). In contrast, the His-SUMO-2B protein oligomerized, and dimer, tetramer and hexamer bands appeared as the glutaraldehyde concentration increased (right side). This result suggests that the purified FMDV 2B protein can oligomerize to form a homomultimer.

A co-immunoprecipitation (co-IP) assay was performed to study the self-interactions of the 2B protein. BHK-21 cells were transfected with the pXJ-FLAG-2B and pXJ-2B-HA plasmids, simultaneously or alone, and collected at 12 hours post-transfection for co-IP. Fig 6B shows that the FLAG tag and the HA tag were simultaneously detected in the cells transfected with the pXJ-FLAG-2B and pXJ-2B-HA plasmids, regardless of whether co-IP was performed. This result suggests that the 2B protein monomers interacted with each other after expression in BHK-21 cells.

### Changes in the Ca^2+^ concentration in BHK-21 cells

**Fig 7:**
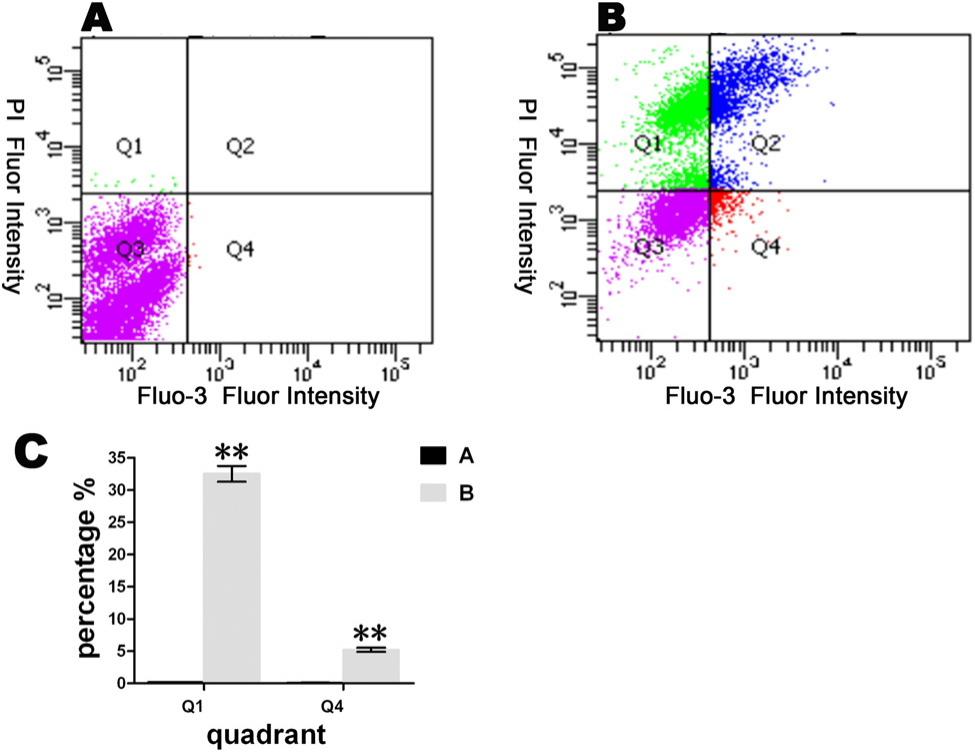
The effects of the 2B protein on the Ca^2+^ content and membrane integrity in host cells. Untreated BHK-21 cells (A) and cells transfected with pXJ-2B-HA (B) were stained with Fluo-3 AM and propidium iodide (PI) at 12 hours post-transfection. The increase in intracellular Ca^**2+**^ (Fluo-3 AM) is shown in Q4. This increase was associated with a change in the PI in BHK-21 cells. A histogram was constructed to reveal the changes in the intracellular Ca^**2+**^ concentration (C). Asterisks indicate significant differences between the indicated samples (*P<0.05,** P<0.01).

The significant effects of viroporins on host cells include disruption of the Ca^2+^ balance [7, 14, 38] and increased plasma membrane permeability [10, 35, 39]. Therefore, the concentration of Ca^2+^ in BHK-21 cells that express the 2B protein was measured using Fluo-3 AM [40], a fluorescence indicator of intracellular Ca^2+^. Simultaneously, PI was used to detect changes in membrane integrity [16, 40]. In Fig 7, the Q1 quadrant represents the fluorescence intensity of PI, which indirectly indicates the amount of membrane damage. The Q4 quadrant represents the fluorescence intensity of Fluo-3, which indirectly indicates the concentration of Ca^2+^. The fluorescence intensity in the Q1 quadrant increased from 0.2% (Fig 7A) to 32.5% (Fig 7B), and the fluorescence intensity in the Q4 quadrant increased from 0.1% (Fig 7A) to 5.2% (Fig 7B). These results suggest host 2B protein expression disrupts membrane integrity and increases the Ca^2+^ concentration in the cytoplasm. The data from the Q1 and Q4 quadrants shown in both Fig 7A and 7B are also shown in a histogram for a better comparison of the changes in the Ca^2+^ content (Fig 7C).

### Autophagy induced by the 2B protein in BHK-21 cells

**Fig 8:**
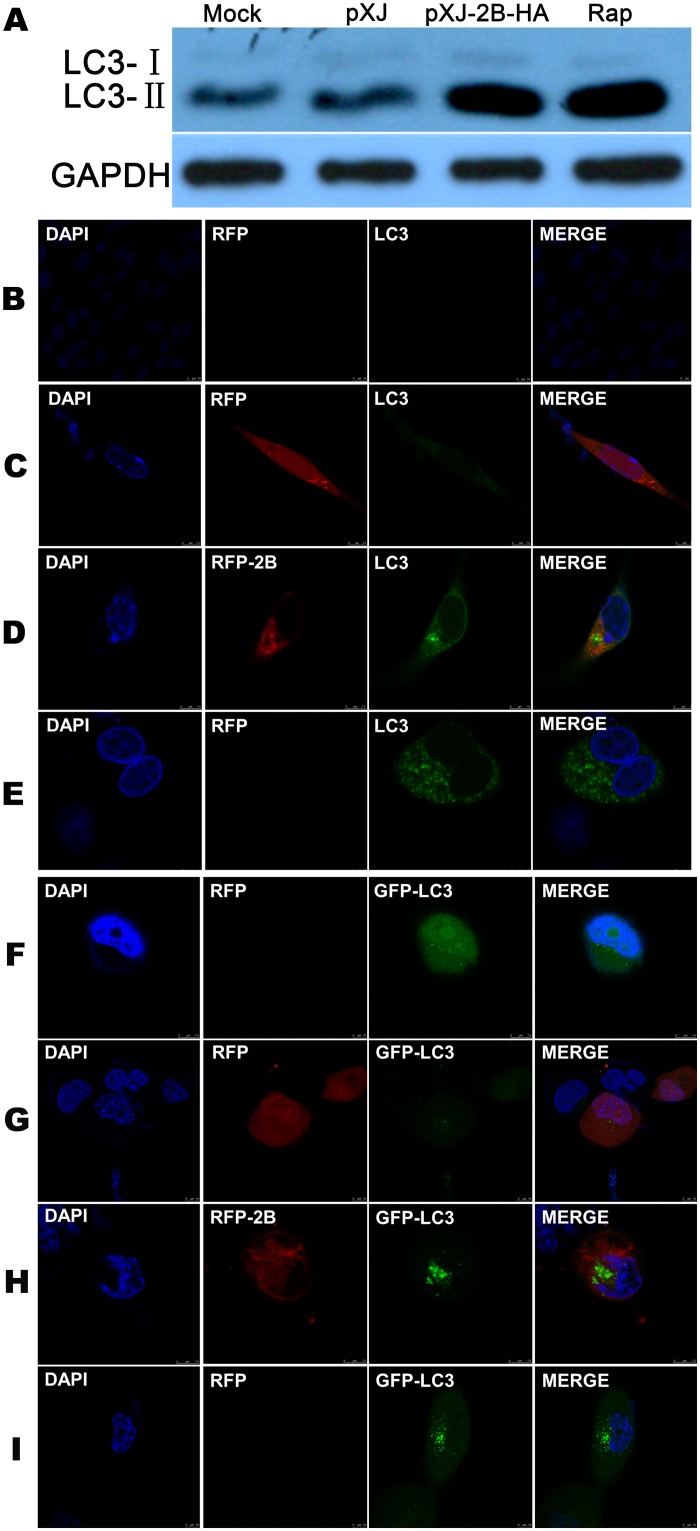
Autophagy induced by the 2B protein in BHK-21 cells and H1299 cells. Four groups of BHK-21 cells were cultured with different treatments, and the LC3-I and LC3-II levels were determined by Western blot analysis with an anti-LC3 antibody (A). Untreated cells (B, F), cells transfected with pCS2 (C, G) or pCS2-2B (D, H), or treated with rapamycin (E, I) were cultured and observed under a LSCM to evaluate LC3 aggregation using IFA in BHK-21 cells (B-E) or based on the green and red fluorescence in H1299 cells (F-I). The blue fluorescence represents the nucleus, the green fluorescence represents the LC3 protein, and the red fluorescence represents the RFP-2B protein.

Many cellular changes, such as changes in the Ca^2+^ content, result in autophagy[41–43]. Viroporins, such as the IAV M2 protein, the rotavirus non-structural protein 1 (NSP1) and NSP4, and the Flavivirus non-structural protein 4A (NS4A), can induce cell autophagy [6, 44–46]. A study on the NSP4 protein further suggested that autophagy is associated with changes in the Ca^2+^ content [6]. As our results demonstrated that the FMDV 2B protein increases the Ca^2+^ content in cells, it would be interesting to determine whether 2B protein expression in host cells induces autophagy by detecting the lipidation of LC3-I to produce LC3-II and the punta formation of LC3 in cells [47]. Cells that were not treated or that were transfected with the pXJ vector were used as a negative control. Cells treated with rapamycin were used as a positive control. All of the cells were collected at 12 hours post-transfection to determine the LC3-I and LC3-II levels by Western blot analysis. Fig 8A shows that the LC3-II band obtained from the cells transfected with pXJ-2B-HA was thick, similar to the band corresponding to the treatment with rapamycin, which is an inducer of autophagy.

The punta formation of LC3 in BHK-21 cells was observed using IFA. Cells that were not treated or were transfected with pCS2, which expresses red fluorescence protein (RFP), were cultured as a negative control. Cells that were treated with rapamycin were used as a positive control. All of the cells were collected at 12 hours post-transfection for fluorescence observation using a LSCM. Fig 8 shows that a low level of LC3 puncta formation was present in both the untreated cells (Fig 8B) and the cells transfected with pCS2 (47), indicating a basal level of autophagy induction in the negative control (Fig 8C). However, significantly more LC3 puncta (238, p = 0.000, p<0.01) were found in the cells transfected with pCS2-2B (Fig 8D), which was similar to the results observed in the positive control (263, p = 0.000, p<0.01) (Fig 8E). Thus, consistent with the Western blot data, the IFA result suggests that the 2B protein activates autophagy induction in BHK-21 cells.

### Autophagy induced by the 2B protein in H1299 cells

To eliminate false-positive results in the IFA, the puncta formation of GFP-LC3 reporter in H1299 cells induced by the 2B protein was evaluated using a LSCM. No significant GFP-LC3 puncta formation was observed in the untreated cells (6) (Fig 8F) or the cells transfected with pCS2 (5) (Fig 8G), which is consistent with the results obtained in the BHK-21 cells. In contrast, GFP-LC3 puncta formed extensively in the cells transfected with pCS2-2B (54, p = 0.000, p<0.01)(Fig 8H) and in the cells used as a positive control (73, p = 0.000, p<0.01)(Fig 8I). These results indicate that only the FMDV 2B protein induced autophagy in the host cells after expression.

## Discussion

Viroporins disrupt the cellular membrane integrity, leading to an increase in permeability. This type of activity has been reported for many viruses. Viroporins can form hydrophilic pores in biological membranes [5]. Additionally, they can also disturb cellular Ca^2+^ homeostasis, induce autophagy, and cause apoptosis [4, 48]. Thus, viroporins enhance the release of viral progeny. In the *Picornavirus* family, the 2B proteins of poliovirus and coxsackie virus, which belong to the *Enterovirus* genus, have been extensively studied as viroporins [38, 49–51]. However, few studies have been conducted on the 2B protein of FMDV, which belongs to the *Aphthovirus* genus of the *Picornaviridae* family of viruses.

In the last several years, significant advances have increased our understanding of the viroporin architecture. Viroporins are classified into two major groups, class I and class II, depending on whether they contain one or two transmembrane domains [5]. Class I viroporins contain only one transmembrane domain and can be further divided into two subgroups: class IA viroporins, with N-termini that face the organelle lumen and C-termini that face the cytoplasmic matrix, and class IB viroporins, with N-termini that face the cytoplasmic matrix and C-termini that face the organelle lumen. Class II viroporins contain two transmembrane domains and are further divided into two subgroups: class IIA viroporins, with both the N-terminus and C-terminus extending into the organelle lumen, and class IIB viroporins, with both the N-terminus and C-terminus extending into the cytoplasmic matrix. In this study, the FMDV 2B protein was predicted to have two hydrophobic regions and was located in the ER. Additionally, this protein exhibited a transmembrane topology similar to that of class IIB viroporins. These results suggest that this protein shares characteristics with viroporins. However, a definitive identification of the domains and function of the two transmembrane regions requires further study.

The solubility and purity of a protein are two important aspects in biochemical and structural analyses of proteins [52], in which the expression of the protein is the first step. We intended to study the pore-forming activity of the FMDV 2B protein in the *Escherichia coli* BL21(DE3)pLysS strain. However, it is difficult to express the FMDV 2B protein in a prokaryote because of the high hydrophobicity of the protein. Fortunately, SUMO fusion technology overcomes this obstacle [52]. The addition of SUMO to the N-terminus of the protein of interest enhances their solubility [53–56]. We intended to confirm the toxicity of the FMDV 2B protein in the *Escherichia coli*BL21(DE3)pLysS strain [8], but the 2B protein was not expressed in this strain. Fortunately, the toxic effect of the 2B protein was confirmed by comparing the BL21(DE3)pLysS cells with the C43(DE3)pLysS cells.

Amantadine is an effective inhibitor of viroporins, such as the IAV M2 protein and the HCV p7 protein. It is well established that FMDV infection is highly sensitive to weak bases that increase endosomal pH. Treatment with amantadine may result in alkalinization of endosomes where FMDV pH-mediated uncoating takes place, thus inhibiting infection. To avoid the pH increase caused by amantadine, we used 1-amantadine hydrochloride instead of amantadine to maintain a weakly acidic pH. Because amantadine inhibited the replication of FMDV, we speculated that amantadine abolished the pore-forming activity of the 2B protein. To verify this proposal, the interactions between 2B protein monomers were analyzed in cells and *in vitro*. Monomer interactions, which may contribute to the pore-forming property of this protein, were detected. In the previous experiment, 2B protein was detected at 2–2.5 h post-infection by immunofluorescence staining. However, no 2B protein expression was detected at different times post-transfection with plasmid pRSV-2B, neither by Western blotting nor by immunofluorescence. This difference may due to cell toxicity. The detection of transient 2B expression has been reported using a modified 2B that included a tag motif [57]. The results were consistent with our findings. We found that the 2B protein changed the cell morphology from fusiform to round and that the cells subsequently died (data not shown), most likely due to the pore-forming activity of the protein. A TEM assay may be needed to further evaluate the ion channel formed by the 2B protein [58].

Many studies on viroporins, including poliovirus 2B and coxsackie virus 2B, have found that these proteins induce Ca^2+^ abnormalities [7]. The flow cytometry analysis indicated that the FMDV 2B protein damaged the membrane integrity and disrupted the Ca^2+^ concentration in host cells, similar to the effects of other viroporins.

Ca^2+^ is one of the most universal and versatile signaling molecules and is involved in most cellular processes [59], including the autophagic pathway. Autophagy is a necessary balancing process, which degrades and reuses intracellular components in response to nutritional deficiencies and other stresses, including viral infection [60, 61]. This study demonstrated that autophagy was upregulated in cells that expressed the FMDV 2B protein at high levels. The disrupted Ca^2+^ levels may have contributed to the increase in autophagy. Adenosine 5’-monophosphate (AMP)-activated protein kinase (AMPK) is a critical protein kinase that induces autophagy, and the phosphorylation of AMPK is regulated by Ca^2+^. An increased Ca^2+^concentration first activates Ca^2+^/calmodulin dependency kinase kinase beta (CaMKK-β), an upstream regulator of AMPK. Then, CaMKK-β induces AMPK phosphorylation, and autophagy occurs [62, 63]. The role of the FMDV 2B protein in this process should be further investigated. In summary, the findings of this study suggest that the FMDV 2B protein exhibits viroporin-like properties and may play an important role in FMDV infection. Additional studies using novel technologies are needed to elucidate the mechanism of action of the FMDV 2B protein.
